# Functional expression of heteromeric insect/human nicotinic acetylcholine receptor chimeras without accessory proteins

**DOI:** 10.1080/19336950.2026.2736911

**Published:** 2026-10-21

**Authors:** Gonxhe Lokaj, Annette Nicke

**Affiliations:** Walther Straub Institute of Pharmacology and Toxicology, Faculty of Medicine, LMU Munich, Munich, Germany

**Keywords:** Insect nicotinic acetylcholine receptor, chimeras, two-electrode voltage-clamp, Xenopus laevis, neonicotinoids

## Abstract

Nicotinic acetylcholine receptors (nAChRs) are primary targets of insecticides. While most mammalian nAChRs form functional channels in heterologous systems, insect nAChRs remain notoriously difficult to reconstitute. Here, we co-expressed insect/human subunit chimeras to enable functional analysis of heteromeric insect nAChR ligand‑binding sites in *Xenopus laevis* oocytes. First, EGFP-tagged human/cockroach α7 chimeras (Hα7/Pα7) were generated by exchanging the signal peptide (SP), extracellular (ECD), transmembrane (TMD), and intracellular (L3-4) domains. While all constructs were translated into protein, Hα7-like acetylcholine-evoked currents were only observed when human N- and C-terminal ECD and ICD were present, indicating that these domains impose insect-specific constraints on homomeric nAChR formation. Second, we generated heteromeric chimeric receptors by fusing insect-derived ECDs onto hα7 subunits. Co-expression of Pα6/Hα7_L3-4_ and Pα7_ECD_/Hα7 chimeras resulted in current amplitudes < 1 µA. Co-expression of the respective bee Aα5_ECD_/Hα7, Aα6_ECD_/Hα7, and Aα7_ECD, CT_/Hα7 chimeras yielded currents > 1 µA. α-Bungarotoxin inhibited diheteromeric Pα6α7 chimeras (IC_50_ 49 nM) and triheteromeric Aα5/α6/α7 chimeras (IC_50_ 51.2 nM). Third, this approach was applied to cockroach Pα2 and Pβ1 subunits. Replacement of the Pα2 ICD and the Pβ1 TMD/ICD with homologous regions from Hα2 and Hβ2 subunits and co-expression with Pα1 resulted in a ~ 100-fold current increase (2.6 µA) compared to the wild‑type Pα1α2β1 receptor. The chimeric receptor was activated by neonicotinoids (EC_50_ clothianidin: 24.1 nM, EC_50_ thiacloprid: 13.6 nM) and α-bungarotoxin-sensitive (IC_50_: 36.6 nM). In conclusion, we demonstrate that replacement of insect ICD or ICD and TMD enables formation of functional insect-like heteromeric nAChRs without co-expression of chaperones.

## Introduction

Nicotinic acetylcholine receptors (nAChRs) are pentameric members of the Cys-loop ligand-gated ion channel family that mediate fast cholinergic neurotransmission in both vertebrates and invertebrates [1–4]. In insects, neuronal nAChRs constitute a primary molecular target of different insecticide classes [5], including spinosyns [6], mesoionic insecticides [7], and the neonicotinoids [8], a major class of insecticides that play a central role in agricultural pest control and public health [9]. Human nAChRs are assembled as homo- or heteropentamers from 16 homologous subunits (α1-α7, α9, α10, β1-β4, δ, γ, ε). Subunits are classified dependent on the presence (α) or absence (β) of adjacent cysteine residues in loop C of the extracellular intersubunit ligand-binding domain. At least one α subunit is essential for ACh coordination. The specific combination of α and β subunits determines receptor properties [10–12]. Insect genomes typically encode 10–12 homologous nAChR subunits, including α1–α7, α8, β2, and β1, found across diverse insect taxa [13,14]. Comparative analyses of genomes from the malaria mosquito, honeybee, silkworm, fruit fly, and red flour beetle (*A. gambiae, A. mellifera, B. mori, D. melanogaster*, and *T. castaneum*) identified seven conserved core groups of nAChR subunits (with β1, β2, α1, α2, α3, α4, and α5–α7 representing different groups) and at least one species-specific divergent subunit, which are supposed to represent promising targets for specific insecticides. Among the seven insect core subunits, α1–α4 and β1–β2 display the highest sequence identity (28–40%) to the human α2 subunit, whereas α5–α7 are most closely related to the human α7 subunit, exhibiting 42–46% amino acid identity [14].

Functional reconstitution of insect nAChRs in heterologous systems has been a major experimental bottleneck, limiting comparative pharmacological analyses beyond a small number of model species [15–17]. Several approaches are used to overcome these limitations, including co-expression of accessory proteins, co-expression of different receptor subunits, and expression of chimeric subunits. Chaperones like RIC-3, UNC-50, TMX-3, and NACHO play an important role in the folding, maturation, and trafficking of cation-selective Cys-loop receptors and have been widely used to improve insect receptor expression [18–24]. The structural determinants governing these interactions have not yet been fully elucidated, but for RIC-3, an interaction with the intracellular loop between TM3 and TM4 of the serotonin receptor was shown [25]. Only a few studies describe the functional expression of homomeric insect subunits [20,26–28]. Interestingly, the Amα5 subunit was shown to form functional channels in *Xenopus laevis* oocytes without accessory proteins, reaching current amplitudes of approximately 2.3 µA. However, receptor activation required high ACh concentrations (EC_50_: 2.4 mM) and serotonin was more potent (EC_50_: 119 µM) [28]. Identification of the *Apis mellifera* chaperone NACHO demonstrated that species-matched accessory proteins can partially overcome barriers, enabling functional expression of homomeric bee α6 receptors that generate small acetylcholine-evoked currents with maximal amplitudes of approximately 15 nA, detectable in roughly 33% of injected oocytes [20]. NACHO from *Drosophila* was more recently shown to enhance functional expression of Dα1Dβ1 nAChRs that were co-expressed with RIC-3, TMX‑3, and UNC‑50 from Drosophila [29].

Co-expression with vertebrate nAChR subunits also yielded functional receptors in *Xenopus* oocytes, but the likely formation of hybrid insect/vertebrate ligand‑binding domains might limit pharmacological characterization and testing [30–33]. Progress in the functional analysis of insect nicotinic acetylcholine receptors has also been achieved for heteromeric assemblies of the model organism *Drosophila melanogaster*. Co-expression of Dα5, Dα6, and Dα7 together with the molecular chaperone RIC-3 in *Xenopus laevis* oocytes yielded a functional receptor with a maximal current amplitude of ~150 nA [27]. However, co-expression of multiple accessory proteins requires optimization of experimental conditions, and current responses are often variable. Also, strategies optimized for *Drosophila* have been difficult to reproduce with closely related α7-like subunits from other species [18,34], indicating the presence of species-specific structural constraints that are not overcome by the commonly used accessory proteins.

More recently, robust functional expression has been achieved for non-α7-like subunits. The Dα1β1 subunit combination, produced reproducible acetylcholine-evoked currents in the range of 0.3–1 µA when co-expressed with chaperones including RIC-3, UNC-50, and TMX-3. Remarkably, incorporation of the Dα2 subunit (Dα1α2β1) resulted in an approximately 50-fold increase in current amplitude to ~20 µA in response to 1 mM acetylcholine (ACh) [22,35].

Chimeric receptor approaches offer a powerful means to overcome challenges in expression. By exchanging domains with those from homologous receptors with favorable expression or functional properties, chimeras have enabled detailed structure–function analyses of nAChRs and other Cys-loop receptors that are otherwise difficult to analyze [36–41]. So far, few studies describe the expression of insect/mammalian nAChR chimeras [42,43]. In the present study, we tested whether a combination of a chimeric approach together with co-expression of different insect subunits is suitable to overcome expression barriers of insect nAChRs. By combining insect ligand‑binding domains with mammalian receptor domains, we generated functional heteromeric α7-like and α1α2β1 chimeric receptors in *Xenopus laevis* oocytes without co-expression of chaperones.

## Results

### Protein translation and functional validation of EGFP-tagged human/cockroach α7 chimeras in *Xenopus laevis* oocytes

In contrast to the human α7 nAChR (Hα7) subunit, which is able to form functional homomeric channels, the *Periplaneta americana* α7 nAChR (Pα7) does not form functional channels on its own. To identify insect nAChR domains that impair channel formation, we generated nAChR subunit chimeras, in which the following domains were swapped between the Hα7 subunit and the non-functional Pα7 subunit: signal peptide (SP, Hα7/Pα7_SP_), N-terminal extracellular domain (ECD, Hα7/Pα7_ECD_), transmembrane domains (TMDs, Hα7/Pα7_TMD_), C-terminal domain (CT, Hα7/Pα7_CT_), and the intracellular TM3–TM4 loop (L3-4), which shows the lowest homology between both receptors (Hα7/Pα7_L3-4_, Figure 1A, Suppl. Figure 1). Two-electrode voltage-clamp analysis of cRNA-injected *Xenopus laevis* oocytes confirmed the formation of homomeric Hα7 receptors with robust acetylcholine-evoked currents in the 1–4 µA range (compare Table 1), whereas Pα7 did not yield any current responses. To assess whether chimeric Hα7/Pα7 subunits are efficiently translated and assembled into functional ion channels, constructs were C-terminally fused to enhanced green fluorescent protein (EGFP), and receptor expression and function were evaluated by SDS–PAGE/fluorescence scanning and two-electrode voltage-clamp (TEVC) recordings (Figure 1A–C). The EGFP-tag did not affect receptor function, as Hα7 and Hα7-EGFP exhibited comparable current kinetics and amplitudes in the 4 µA range. SDS–PAGE analysis revealed a double band, likely representing core- and complex-glycosylated forms, with a lower band at approximately ~70 kDa and an upper band at ~75 kDa (Figure 1A). Among the chimeras, only Hα7 constructs containing the insect signal peptide (Hα7/Pα7_SP_) and transmembrane domains (Hα7/Pα7_TMD_) showed a clear band at ~75 kDa, whereas all the other chimeras displayed either no detectable band or weaker bands of intermediate size. In agreement with these biochemical data, TEVC recordings (Figure 1B,C) of ACh-evoked peak currents revealed robust currents in the 2–9 µA range for chimeras containing insect signal peptide and transmembrane domains, indicating that these regions are compatible with functional receptor assembly.

**Figure 1.**
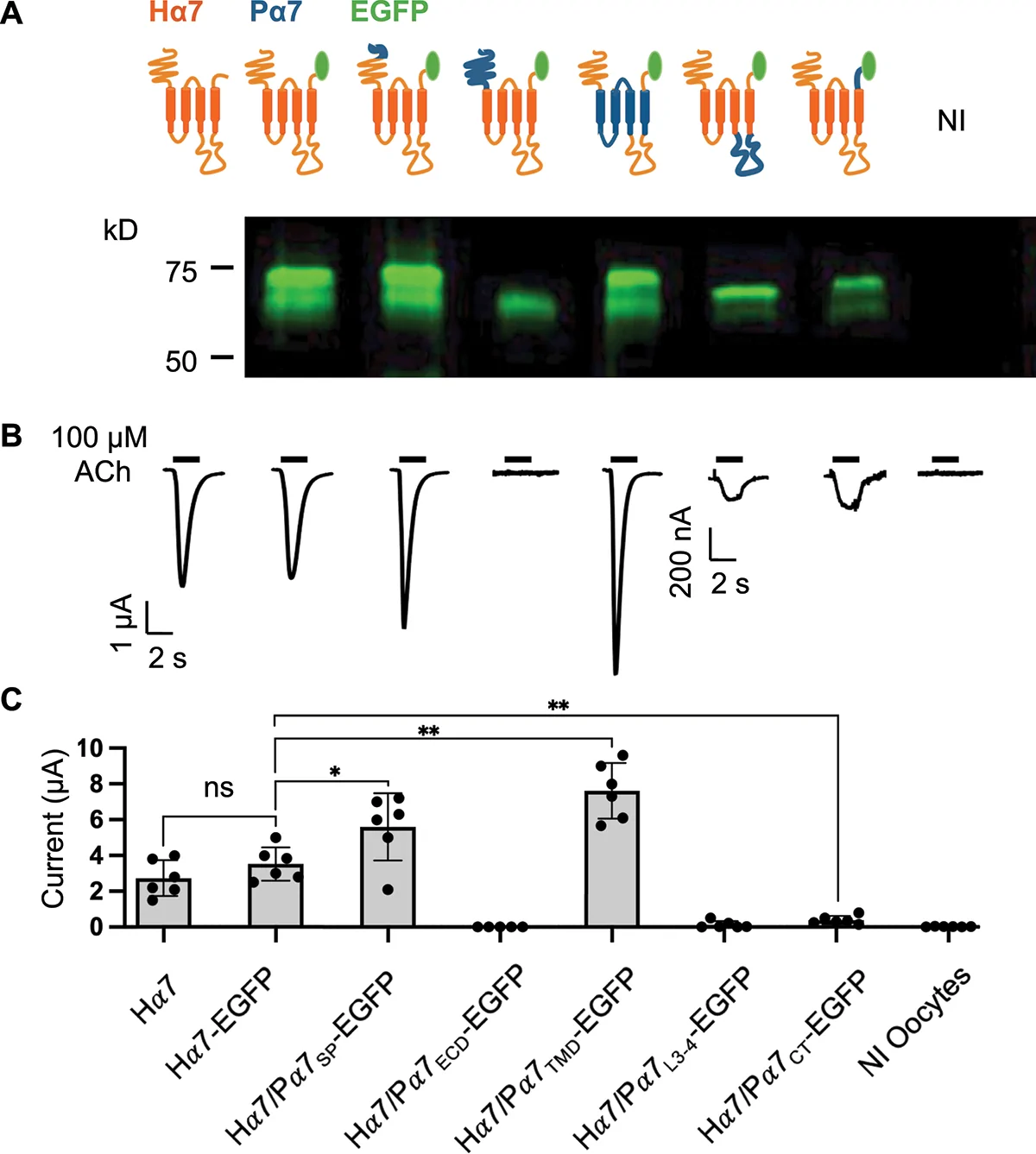
Protein translation and functional validation of EGFP-tagged human/cockroach chimeras in *Xenopus laevis* oocytes. (A) Schematic representation of human α7 and chimeric constructs generated by domain exchange between human α7 (orange) and *Periplaneta americana* α7 (blue, EGFP-tags are indicated in green) and a representative fluorescence scan of the indicated nAChR subunits separated by SDS-PAGE (8% gel). Proteins were extracted in dodecylmaltoside, purified via Ni-NTA agarose, and amounts equivalent to four oocytes were loaded on the gel. (B) Representative current traces recorded from the indicated chimeras by two-electrode voltage clamp at −70 mV. (C) Comparison of ACh-evoked peak current amplitudes of the indicated receptors. Data are shown as mean ± SD, with recordings from individual oocytes represented as dots. Statistical significance was assessed using an unpaired Welch’s t-test (*p < 0.05, **p < 0.01). *n* = 6 oocytes per receptor from at least 2 batches. NI = non-injected.

**Table 1.** Summary of current amplitudes and pharmacological properties of native and chimeric insect/human nAChRs expressed in *Xenopus laevis* oocytes.

Chimeras	*I*_peak_ (μA)	α-Btx sensitivity		Agonist sensitivity	
		IC_50_ (nM)	*n*_H_	EC_50_ (nM)	*n*_H_
Hα7	2.73 ± 1	n.d.		n.d.	
Hα7-EGFP	3.52 ± 0.93	n.d.		n.d.	
Hα7/Pα7_SP_-EGFP	5.6 ± 1.89	n.d.		n.d.	
Hα7/Pα7_ECD_-EGFP	–				
Hα7/Pα7_TMD_-EGFP	7.56 ± 1.58	n.d.		n.d.	
Hα7/Pα7_L3-4_-EGFP	0.35 ± 0.22	n.d.		n.d.	
Hα7/Pα7_CT_-EGFP	0.43 ± 0.28	n.d.		n.d.	
Pα7	–				
Hα7_ECD_/Pα7	–				
Pα6/Hα7_L3-4_	–				
Pα7/Hα7_L3-4_	–				
Pα6/Hα7_L3-4_ Pα7/Hα7_L3-4_	–				
Pα6/Hα7_L3-4_ Hα7/Pα7_ECD_	0.20 ± 0.07	49.0 [38.1–57.9]	−1.5 [−1.9–1.2]	n.d.	
Aα5_ECD_/Hα7	–				
Aα6_ECD_/Hα7	–				
Aα7_ECD_/Hα7	–				
Aα7/Hα7_ECD_	–				
Aα7/Hα7_L3-4,CT_	–				
Aα7/Hα7_L3-4,TMD4,CT_	–				
Aα7/Hα7_TMD2-3,L3-4,CT_	–				
Aα7/Hα7_TMD1-3,L3-4,CT_	–				
Aα7_L3-4_/Hα7	–				
Aα7_ECD ,CT_/Hα7	–				
Aα5_ECD_/Hα7 Aα6_ECD_/Hα7	–				
Aα5_ECD_/Hα7 Aα7_ECD_/Hα7	–				
Aα5_ECD_/Hα7 Aα7/Hα7_ECD_	–				
Aα5_ECD_/Hα7 Aα7/Hα7_L3-4,CT_	–				
Aα5_ECD_/Hα7 Aα7/Hα7_L3-4,TMD4,CT_	–				
Aα5_ECD_/Hα7 Aα7/Hα7_TMD2-3,L3-4,CT_	–				
Aα5_ECD_/Hα7 Aα7/Hα7_TMD1-3,L3-4,CT_	–				
Aα5_ECD_/Hα7 Aα7 _L3-4_/Hα7	–				
Aα5_ECD_/Hα7 Aα7 _ECD, CT_/Hα7	–				
Aα6_ECD_/Hα7 Aα7_ECD_/Hα7	–				
Aα6_ECD_/Hα7 Aα7/Hα7_ECD_	–				
Aα6_ECD_/Hα7 Aα7/Hα7_L3-4,CT_	–				
Aα6_ECD_/Hα7 Aα7/Hα7_L3-4,TMD4,CT_	–				
Aα6_ECD_/Hα7 Aα7/Hα7_TMD2-3,L3-4,CT_	–				
Aα6_ECD_/Hα7 Aα7/Hα7_TMD1-3,L3-4,CT_	–				
Aα6_ECD_/Hα7 Aα7_L3-4_/Hα7	–				
Aα6_ECD_/Hα7 Aα7_ECD, CT_/Hα7	–				
Aα7_ECD_/Hα7 Aα7/Hα7_ECD_	–				
Aα7_ECD_/Hα7 Aα7/Hα7_L3-4,CT_	–				
Aα7_ECD_/Hα7 Aα7/Hα7_L3-4,TMD4,CT_	–				
Aα7_ECD_/Hα7 Aα7/Hα7_TMD2-3,L3-4,CT_	–				
Aα7_ECD_/Hα7 Aα7/Hα7_TMD1-3,L3-4,CT_	–				
Aα7_ECD_/Hα7 Aα7_L3-4_/Hα7	–				
Aα7_ECD_/Hα7 Aα7_ECD, CT_/Hα7	–				
Aα7/Hα7_ECD_ Aα7/Hα7_L3-4,CT_	–				
Aα7/Hα7_ECD_ Aα7/Hα7_L3-4,TMD4,CT_	–				
Aα7/Hα7_ECD_ Aα7/Hα7_TMD2-3,L3-4,CT_	–				
Aα7/Hα7_ECD_ Aα7/Hα7_TMD1-3,L3-4,CT_	–				
Aα7/Hα7_ECD_ Aα7_L3-4_/Hα7	–				
Aα7/Hα7_ECD_ Aα7_L3-4,CT_/Hα7	–				
Aα5_ECD_/Hα7 Aα6_ECD_/Hα7 Aα7_ECD_/Hα7	0.45 ± 0.08	n.d.		n.d.	
Aα5_ECD_/Hα7 Aα6_ECD_/Hα7 Aα7/Hα7_ECD_	–				
Aα5_ECD_/Hα7 Aα6_ECD_/Hα7 Aα7/Hα7_L3-4,CT_	0.28 ± 0.14	n.d.		n.d.	
Aα5_ECD_/Hα7 Aα6_ECD_/Hα7 Aα7/Hα7_L3-4,TMD4,CT_	0.18 ± 0.05	n.d.		n.d.	.
Aα5_ECD_/Hα7 Aα6_ECD_/Hα7 Aα7/Hα7_TMD2-3,L3-4,CT_	0.28 ± 0.12	n.d.		n.d.	
Aα5_ECD_/Hα7 Aα6_ECD_/Hα7 Aα7/Hα7_TMD1-3,L3-4,CT_	0.26 ± 0.03	n.d.		n.d.	
Aα5_ECD_/Hα7 Aα6_ECD_/Hα7 Aα7_L3-4_/Hα7	–				
Aα5_ECD_/Hα7 Aα6_ECD_/Hα7 Aα7_ECD, CT_/Hα7	1.57 ± 0.22	51.2 [47–68.8]	−1.3 [−1.6–1.0]	n.d.	
Pα1	–				
Pβ1	–				
Pα2	–				
Pα2/Hα2_L3-4_	–				
Pβ1_ECD_/Hβ2	–				
Pα1	–				
Pβ1	–				
Pα2	–				
Pα2/Hα2_L3-4_	–				
Pβ1_ECD_/Hβ2	–				
Pα1 Pβ1	–				
Pα1 Pα2	–				
Pα1 Pα2/Hα2_L3-4_	–				
Pα1 Pβ1_ECD_/Hβ2	–				
Pα2 Pβ1	–				
Pα2/Hα2_L3-4_ Pβ1	–				
Pα2 Pβ1_ECD_/Hβ2	–				
P2/Hα2_L3-4_ Pβ1_ECD_/Hβ2	–				
Pα1 Pα2 Pβ1					
Pα1 Pα2/Hα2_L3-4_ Pβ1	0.34 ± 0.08	n.d		n.d	
Pα1 P2/Hα2_L3-4_ Pβ1_ECD_/Hβ2	2,42 ± 0.31	36.6 [27 – 48.3]	-1.0 [-1.3 – 0.8]	A 2394 [1876 – 3051] C 24.1 [11.0 – 40.3] T 13.6 [10.1 – 18.4]	-1.0 [-0.8 –1.4] -0.9 [-05 – -1.4] -0.7 [-0.6 – 0.9]

Current amplitudes (*I*_peak_) are given as mean ± SD. Numbers in square brackets indicate 95% confidence intervals. α-Btx = α-Bungarotoxin; A = Acetylcholine; C = Clothianidin; T = Thiacloprid; *n*_H_ = Hill coefficient; n.d. = not determined. Groups of chimeras are separated according to paragraphs in the results part and associated figures. Values below 100 nA were not considered.

In contrast, substitution of the Hα7 ECD or the L3-4 intracellular loop with the corresponding Pα7 regions resulted in nonfunctional receptors or only minimal current amplitudes in the 400–800 nA range. These findings suggest that key determinants limiting functional expression reside in the Pα7 ECD and the L3-4 intracellular loop. To further test this hypothesis, we generated reverse chimeras, in which the Pα7 ECD and L3-4 were replaced by the corresponding Hα7 domains. However, no functional currents were observed (Table 1), suggesting that a combination of domains or additional insect subunits is required for receptor formation.

### Formation of a α-bungarotoxin-sensitive cockroach α6α7 ligand-binding domain

Given that functional expression of α7-like insect receptors has previously been demonstrated for the most closely related *Drosophila* Dα5Dα6Dα7combination [27] and a Pα5 subunit does not exist, we first tested whether Pα7 requires co-assembly with the Pα6 subunit for functional receptor formation. Initial experiments with wild‑type Pα subunits, either expressed individually or co-expressed as Pα6Pα7, failed to yield detectable functional responses (Figure 2A, compare Table 1). We therefore first substituted the divergent intracellular loop 3–4 in both Pα6 and Pα7 with the corresponding Hα7 sequence (Pα6/Hα7_L3-4_, Pα7/Hα7_L3-4_). However, this also did not result in significant receptor currents (Figure 2A). Thus, we next used the Pα7_ECD_/Hα7 chimera, in which the whole ICD, TM, and CT domains were human and only the ECD was from cockroach (compare Figure 1). Co-expression of this chimera with the corresponding Pα6/Hα7_L3-4_ chimera resulted in small but robust acetylcholine-activated currents with peak amplitudes in the 100–300 nA range (Figure 2A,B). The corresponding “Pα6α7-like receptor” (Pα6/Hα7_L3-4_+Pα7_ECD_/Hα7) was concentration-dependently inhibited by α-bungarotoxin (α-Btx) with an IC_50_ of 49 nM (Figure 2C), in agreement with the α-Btx sensitivity of α7 nicotinic acetylcholine receptors from other species. Thus, phylogeny-guided combination of insect/human subunit chimeras enabled the functional reconstitution of an α-Btx-sensitive cockroach α6α7 ligand‑binding site in a heterologous expression system without co-expression of chaperones. The fact that Pα7_ECD_/hα7 was not functional on its own (compare Figure 1) suggests that it requires heteromeric assembly. Since the α5 subunit is absent in cockroach, we next used honey bee (*Apis mellifera*) subunits to investigate whether a combination of Aα5, Aα6, and Aα7 subunits would lead to higher currents as described for Drosophila [27].

**Figure 2.**
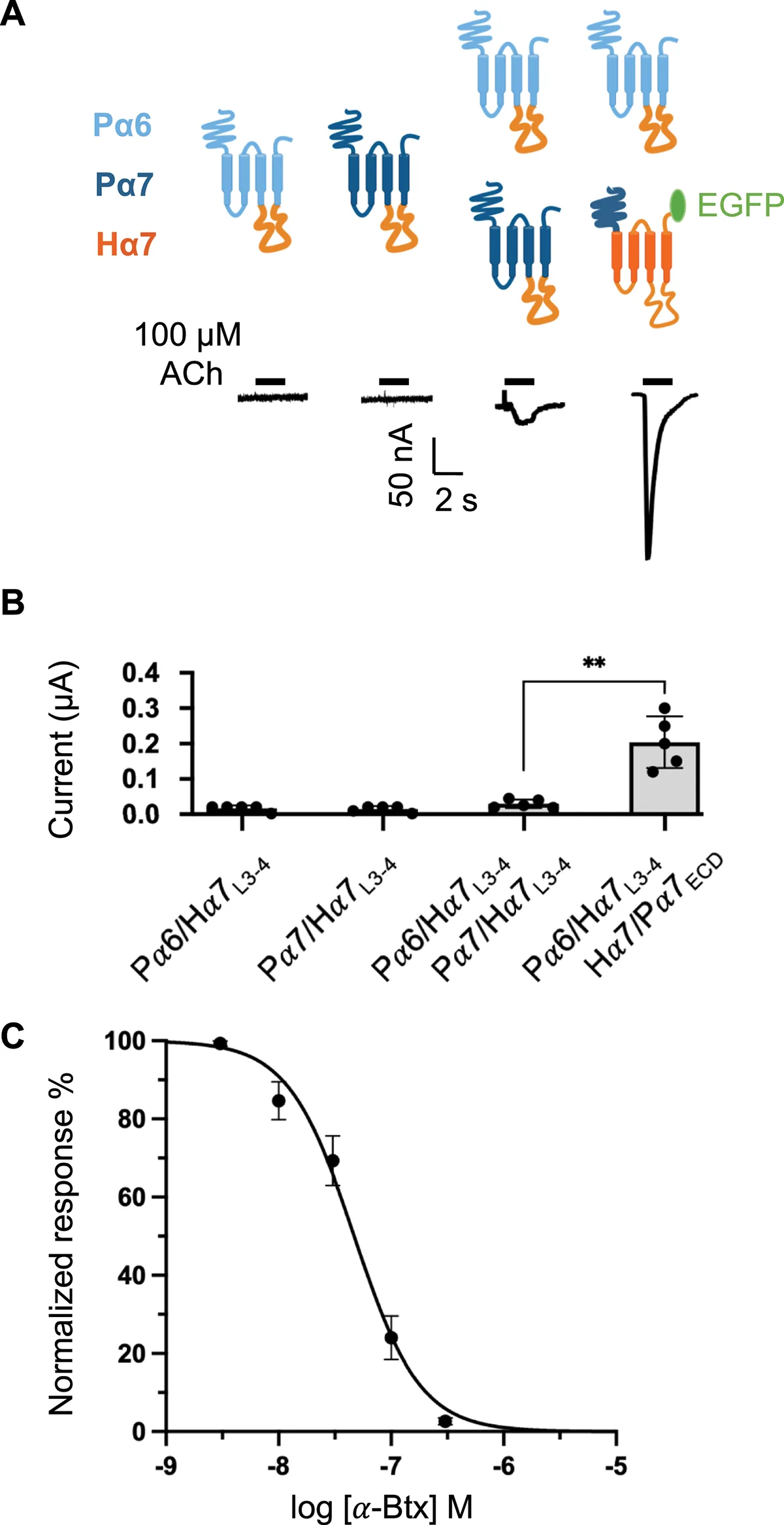
Functional validation of cockroach/human nAChR subunit chimeras in *Xenopus laevis* oocytes. (A) Schematic representation of *P. americana* α6 and α7 (shades of blue) ECDs and TM domains fused to human (orange) α7 loop 3–4 and TM domains and representative ACh-induced current traces recorded from *X. laevis* oocytes expressing the indicated chimeric subunits. Horizontal black bars indicate application of 100 µM ACh. (B) Comparison of ACh-evoked peak current amplitudes of the indicated receptors. TEVC recordings were performed at −70 mV. Data are shown as mean ± SD, with recordings from individual oocytes represented as dots. Statistical significance was assessed using Welch’s t-test *p < 0.05, **p < 0.01). *n* = 6 oocytes per receptor from at least 2 batches. (C) α-BTX dose‑inhibition curve of ACh (100 μM)-evoked currents recorded from the Pα6/Hα7_L3-4_Hα7/Pα7_ECD_heteromeric chimera. Mean ± SEM of *n* = 5 recordings from individual oocytes from at least 2 batches are shown. IC_50_of α-BTX = 49 nM.

### Formation of an α-btx-sensitive bee α5α6α7 ligand-binding domain

So far, our experiments have shown that functional Pα6α7 nAChR ligand‑binding sites can be formed without chaperones by grafting Pα ECD domains on human α7 subunits. To test whether this applies also to other insect species, we next created chimeras of the honey bee *Apis mellifera* Aα5, Aα6, and Aα7 N-terminal ECDs on Hα7 subunits (Aα5_ECD_/Hα7, Aα6_ECD_/Hα7, and Aα7_ECD_/Hα7, Figure 3A). In agreement with previous findings for the fruit fly [27], coexpression of all three chimeras revealed small (<1 μA) but robust ACh-evoked currents (Figure 3A,C), while individual, pairwise, or triplet expression of the wild-type Aα5, Aα6, and Aα7 or individual or pairwise expression of the chimeric α5, α6, and α7 subunits did not yield detectable acetylcholine-evoked currents (compare Table 1). In contrast to [27], however, our chimeric approach did not require co-expression of RIC-3.

**Figure 3.**
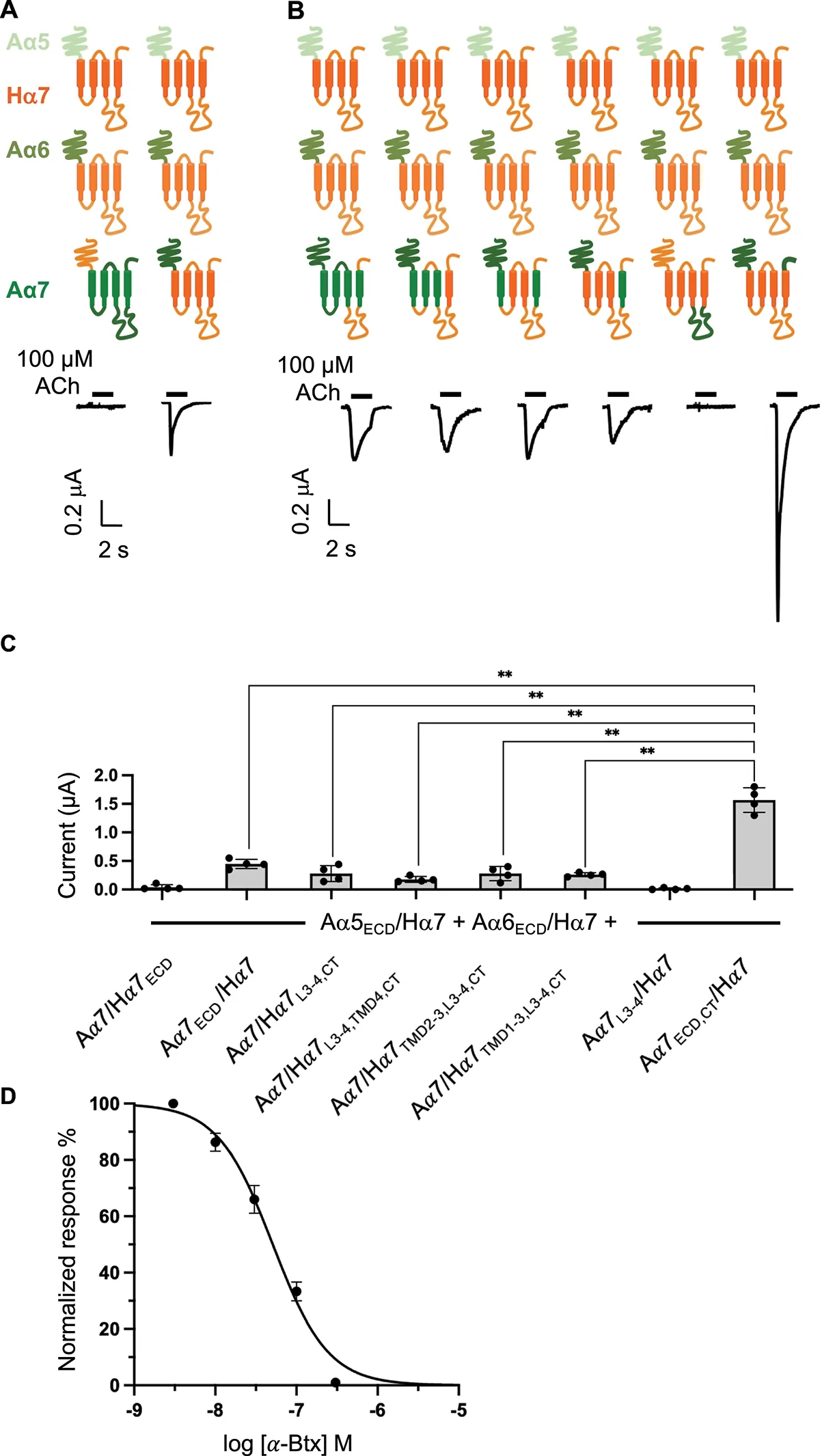
Functional validation of triheteromeric receptors from chimeras of honey bee α5, α6, and α7 ECDs with human α7 in *Xenopus laevis* oocytes. (A) Schematic representation of *A. mellifera* α5, α6, and α7 ECDs (shades of green) fused to human α7 (orange) and (B) Constructs in which distinct additional bee α7 domains were introduced with the corresponding representative ACh-induced current traces and peak amplitudes (C). *X. laevis* oocytes were injected with RNA for different Aα7/Hα7 chimeras together with Aα5_ECD_/Hα7 and Aα6_ECD_/Hα7 chimeras, as indicated in A/B.   TEVC recordings were performed at −70 mV. Data are shown as mean ± SD, with recordings from individual oocytes represented as dots. Statistical significance was assessed using Welch’s t-test (*p < 0.05, **p < 0.01). *n* = 4 oocytes from two batches per receptor combination. (D) Dose‑inhibition curve of α-BTX at the triheteromeric chimeric subunit combination of Aα5_ECD_/Hα7, Aα6_ECD_/Hα7, and Aα7_ECD, CT_/Hα7. Current responses were evoked by 100 μM ACh and TEVC recordings were performed at −70 mV. Mean ± SEM of *n* = 5 individual oocytes from at least 2 batches are shown. IC_50_ for α-BTX = 51.2 nM.

In analogy to our experiments with cockroach/human chimeras (compare 2.1), we next exchanged additional domains in the Hα7 with the respective Aα7 sequences to identify further bee domains that are tolerated in these complexes and make the resulting receptors more “insect-like.” Altogether, six additional Aα7/Hα7 chimeras were generated and co-expressed with the unaltered Aα5_ECD_/Hα7 and Aα6_ECD_/Hα7 chimeras (Figure 3B). Since extracellular N-and C-terminal regions have been involved in receptor assembly and folding [44], these domains were also exchanged in combination. As seen in Figure 3B,C a significant increase in current amplitude (1–2 µA) was only observed when both the Aα7 N- and C-terminal domains were present, suggesting that both appropriate subunit assembly and species-specific interactions between the N- and C-terminal domains are essential for the formation of functional receptors. Further analysis of this receptor revealed again sensitivity to α-Btx with an IC_50_ of 51.2 nM (Figure 3D).

### Formation of α-Btx and nicotinoid-sensitive cockroach α1α2β1 ligand‑binding sites

Building on previous studies demonstrating that incorporation of the Dα2 subunit into the established Dα1β1 receptor results in an approximately 50-fold increase in functional response, if co-expressed with chaperones [35], we investigated whether an analogous result could be obtained with the respective Pα1, Pα2, and Pβ1 subunits, which cluster within the corresponding *Drosophila* α1, α2, and β1 clade (Suppl. Figure 3). Co-expression of the three wild‑type cockroach subunits resulted in very small ACh-evoked currents in the low nanoampere range (Figure 4A). To further enhance the formation of functional receptors, we applied the chimera strategy by replacing the intracellular and TM domains of the Pα1, Pα2, and Pβ1 subunits with the homologous segments of the most closely related Hα2 and Hβ2 nAChR subunits, respectively (Figure 4A). Substitution of the L3-4 loop of Pα2 with the corresponding Hα2 sequence (Pα2/Hα2 L_3–4_) resulted in a current increase to approximately 300 nA (Figure 4B). Additional fusion of the Pβ1 ECD to the respective Hβ2 subunit resulted in an about 20-fold current increase with mean peak amplitudes of 2-3 µA. In contrast, control experiments with single wild‑type and chimeric subunits or dimeric combinations produced no or only minor currents of up to ~20 nA (compare Table 1), comparable to those observed for the wild‑type heterotrimers. Concentration–response analysis of this optimized heterotrimeric “Pα1Pα2Pβ1-like” receptor revealed an EC_50_ of 2.4 µM for ACh (Figure 4C), consistent with previously reported values (5.8–7.8 μM) for heteromeric Dα1Dβ1 and Dα1DαDβ1 nAChRs [22,35].

**Figure 4.**
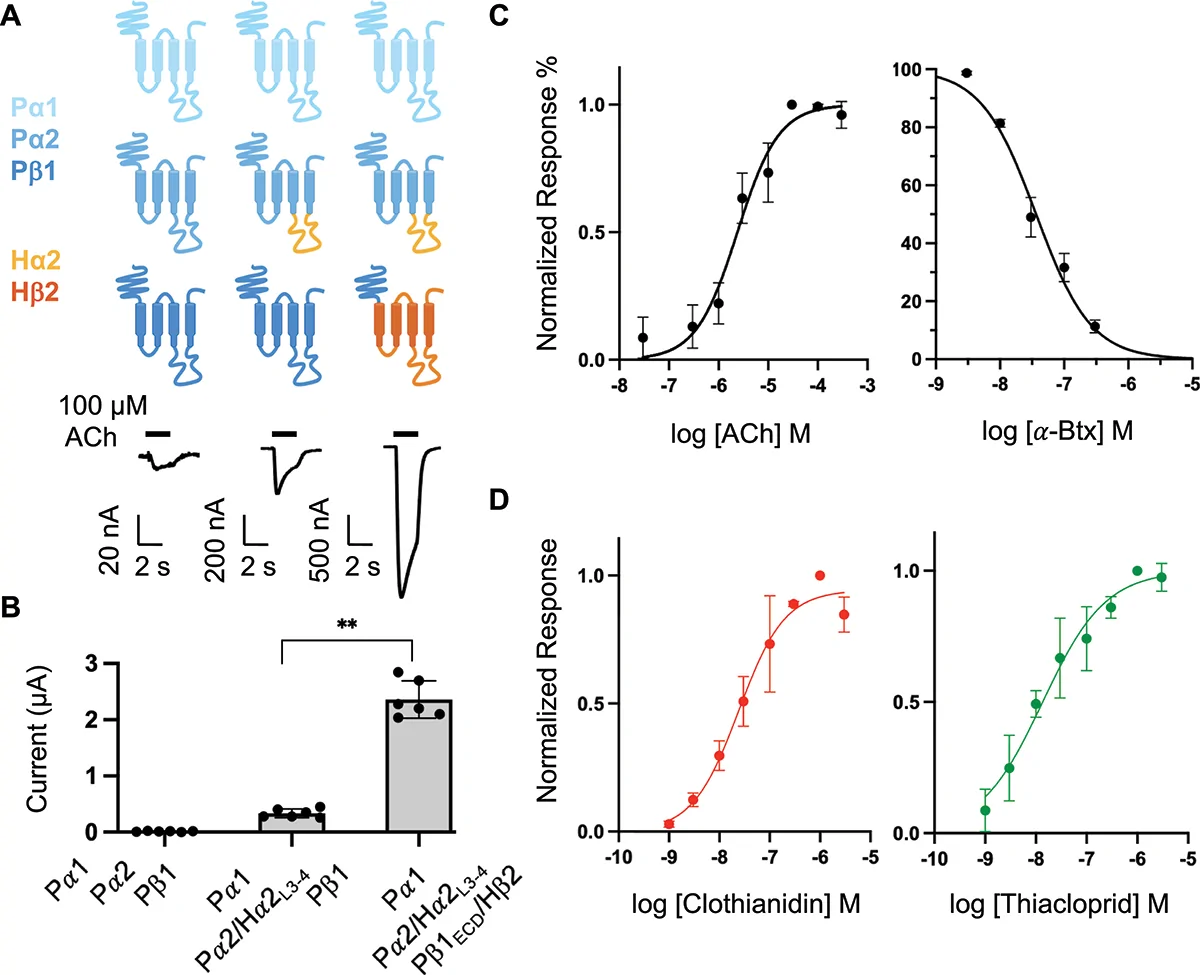
Functional and pharmacological characterization of a heterotrimeric nAChRs from chimeric α1, with chimeric cockroach/human α2/α2, and β1/β2 subunits expressed in *Xenopus laevis* oocytes. (A) Schematic representation of chimeras between human α2 and β2 (shades of orange) and *P. americana* α1, α2, and β1 (shades of blue) subunits generated by domain exchange, and representative ACh-induced currents recorded from *X. laevis* oocytes expressing the indicated triheteromeric chimeric subunit combinations. Horizontal black bars indicate application of 100 μM ACh. (B) Comparison of ACh-evoked peak current amplitudes of the indicated combinations. TEVC recordings were performed at −70 mV. Data are shown as mean ± sd, with recordings from individual oocytes represented as dots. Statistical significance was assessed using Welch’s *t*-test (*p < 0.05, **p < 0.01). *n* = 6 oocytes per receptor from at least 2 batches. (C) Concentration–response relationships for ACh and α-BTX and (D) the neonicotinoids clothianidin and thiacloprid at the triheteromeric nAChR formed by wild-type Pα1 and the Pα2/Hα2_L3-4_ and Pβ1_ECD_/Hβ2 chimeras. Each point represents mean ± SEM (*n* = 6). The calculated IC_50_ for α-BTX = 36.6 nM, EC_50_ for ACh = 2.4 μM, EC_50_ for clothianidin = 24.1 nM, and EC_50_ for thiacloprid = 13.6 nM. Each point represents mean ± SEM of *n* = 6 oocytes from at least two batches. For comparison of variability of current responses between oocyte batches, see Fig. S4.

α-Btx inhibited ACh-evoked currents with an IC_50_ of 36.6 nM (Figure 4C), also in close agreement with values reported from a patch‑clamp study on *Periplaneta americana* neurons, where an IC_50_ of 21 nM for α-Btx and an EC_50_ for ACh of 6.6 μM were determined [45]. Furthermore, the receptor was sensitive to the neonicotinoids clothianidin and thiacloprid, with EC_50_ values of 24.1 nM and 13.6 nM, respectively (Figure 4D), consistent with previously described neonicotinoid-sensitive native insect nAChR and Dα1Dα2Dβ1 subtypes [8,46]. Thus, the replacement of insect nAChR subunit domains that diverge from mammalian nAChR subunits with the respective sequence of a homologous human subunit can improve the formation of functional heteromeric insect nAChRs in a heterologous system without chaperones and exhibit pharmacological properties of native insect receptors.

## Discussion

Expression of functional insect nAChRs in heterologous systems has been highly challenging and frequently results in either absent or variable current responses [18,20]. Most successful approaches to record from recombinant insect receptors relied on co-expression of insect subunit combinations together with mammalian or insect-derived chaperones in *Xenopus laevis* oocytes [18–20,22,24,29,35].

Here, we used an approach based on sequence comparison and chimera formation between homologous mammalian and insect nAChR subunits and demonstrate that functional heteromeric insect nAChR receptor ligand‑binding sites can be efficiently formed in *Xenopus laevis* oocytes without the need of chaperones. Expressing more than 25 wild-type and chimeric nAChR subunits in about 50 different combinations, we identify specific receptor domains and subunit combinations that influence the formation of functional receptors in heterologous systems and provide evidence that 1) introduction of insect SP and TM domains into human subunits is well tolerated while key regions that prevent efficient receptor formation reside in the intracellular L3-4 loop and the ECD; 2) species-specific interactions between extracellular N- and C-termini can be important; and 3) heteromeric assembly of insect α7-related subunits is preferred over homomer formation. The latter was unexpected and is in contrast to mammalian α7 nAChR subunits that can efficiently form homomers in *Xenopus laevis* oocytes but require co-expression of chaperones for efficient expression in cell-lines [47].

Domain-swapping has been widely used in members of the Cys-loop receptor superfamily [36,41] both between and within receptor classes and species. Together, the results show that modular replacement strategies are well tolerated and that chimeric receptor strategies can overcome major limitations in receptor expression and might even help structural analysis. Based on a study on homomeric human/insect α7 chimeras, Cartereau et al. [42] recently proposed a molecular mechanism in which the human Cys-loop mediates functional coupling between insect ECD and human TM/ICD domains in α7 homomeric receptors. Our work demonstrates that, heteromerization, together with replacement of intracellular and TM domains, enables reproducible (compare Fig S4) and robust (2–3 μA) functional expression of insect nAChRs without requiring accessory proteins. A consistent observation in our study was that no single domain replacement was sufficient to restore functional expression. Instead, successful insect receptor reconstitution appears to rely on both additional insect subunits and the replacement of divergent regions that compromise functional expression. In Aα7 subunits, the interplay between extracellular N- and C-terminal domains was also found to be critical, whereas in the heteromeric Paα1Pα2Pβ1 receptor, replacement of intracellular and transmembrane regions was already sufficient for efficient receptor formation. Our results support the hypothesis that both insect ECDs and ICDs affect functional heterologous expression. At the ECD level, receptor expression is hampered if critical assembly partners are missing, and this can be overcome by heteromerization. Likewise, insect L3-4 in the ICD, which differs considerably from the mammalian L3-4, might require insect-specific interactions with accessory proteins for efficient expression, and this can be overcome by domain exchange. Further studies focusing on (1) the roles of insect ECDs in subunit assembly and/or coupling to the channel gate and (2) the effects of L3-4 versus L3-4 plus TMD exchange are required to evaluate the individual contributions of these domains.

Several limitations of the study need to be considered. 1) Due to technical limitations (low expression levels, lack of antibodies), most of the presented experiments do not distinguish directly between effects on translation, folding, trafficking, assembly, or gating. Thus, we cannot be sure that some of our chimeras were not synthesized, misfolded, did not form functional ACh binding sites, or showed gating defects. However, fusion sites were chosen in conserved receptor domains, and the fact that several chimeras formed functional receptors if co-expressed with the appropriate subunits supports our assumption that most chimeras are principally able to fold correctly and form functional receptors.

2) All experiments were performed in *Xenopus laevis* oocytes, which were previously shown to be more efficient in the expression of critical nAChRs than mammalian expression systems [15]. Thus, confirmation of our results in a cell line would be an important future perspective. 3) The subunit combinations were chosen based on homology and do not necessarily reflect native receptor architecture. To further explore suitable subunit assemblies, transcriptomic analysis of different insect cell types might be helpful. 4) The present work focuses on optimization of ACh-evoked current amplitudes as a measure for ligand‑binding site formation and does not address receptor stoichiometry, assembly pathways, or gating kinetics, which might be affected by the chimeric strategy. 5) Finally, the presented chimeric approach is likely restricted to the investigation of ligands that interact with the extracellular orthosteric ligand‑binding site (such as neonicotinoids and mesoionic insecticides) but are not suitable to faithfully study the mode of action of compounds that bind to or act allosterically via the transmembrane domains (such as the spinosyn class of insecticides and the positive allosteric modulator PNU-120596) or open channel blockers. Within these constraints, the results provide a basis for a practical framework to generate functional insect nAChR constructs. To test the general applicability of this approach, we selected two biologically and pharmacologically relevant insect systems: the pest species *Periplaneta americana* and the beneficial pollinator *Apis mellifera*. While general assembly principles may be shared, successful functional reconstitution of specific subtypes might require tailored strategies for each subtype and species. Resulting receptors might be further used to pharmacologically define insect nAChR subtypes and identify species-selective ligands as superior novel pesticides.

## Materials and methods

### cDNA constructs and cRNA synthesis

The nicotinic acetylcholine receptor coding sequences were cloned into the pNKS2 expression vector for *Xenopus laevis* oocyte expression [48], using Gibson assembly [49]. cDNAs encoding nAChR subunits from *Periplaneta americana* α1 (JQ585634), α2 (KP725464), β1 (MW201213), α6 (KP725465), and α7 (MK790056.1); *Apis mellifera* α5 (KJ939592), α6 (DQ026036), and α7 (AY500239.1); and *Homo sapiens* α2 (NM_000742), β2 (NM_000748), and α7 (NM_000746) were used (Suppl. Table 1). Chimeric constructs were generated by Gibson assembly using PCR-amplified inserts and linearized vector backbone fragments with overlapping ends. Primers were designed based on sequence alignments between insect and mammalian nAChR subunits to define chimera junctions and domain boundaries. PCR amplification was performed using high-fidelity Q5 polymerase (New England Biolabs, USA), and assembled plasmids were transformed into *Turbo E. coli* (New England Biolabs, USA). Constructs were endowed with a hexahistidyl sequence, and where indicated, EGFP-tags were fused via a heptahistidyl sequence to the coding sequences. All constructs were verified by full-length sequencing (Eurofins Genomics, Germany). Detailed sequence alignments, chimera designs, and transition regions are provided in Suppl. Figure 2. For cRNA synthesis, plasmids were linearized with *NotI* (New England Biolabs, USA) and transcribed *in vitro* using the SP6 mMESSAGE mMACHINE kit (Invitrogen, Thermo Fisher Scientific, USA) according to the manufacturer’s instructions.

### Biochemical analysis

2–5 d after cRNA injection, 20 oocytes were homogenized in 200 μl ice-cold homogenization buffer containing 0.1 M sodium phosphate buffer (pH 8.0), 1% dodecylmaltoside (DDM, Carl Roth), and protease 0.2 mM Pefabloc® SC protease inhibitor (Sigma-Aldrich), incubated on ice for 15 min, and clarified by two centrifugation steps (10 min, 14,000 × g, 4°C). His-EGFP-tagged receptor proteins were enriched using Ni-NTA agarose beads (Qiagen). Prior to use, the beads were equilibrated three times with wash buffer consisting of 0.1 M sodium phosphate buffer (pH 8.0) supplemented with 25 mM imidazole and 0.2% DDM. 200 μl of membrane extracts were diluted with 300 μl homogenization buffer and incubated with 30 μl of Ni-NTA beads for 1 h at 4°C on an end-over-end mixer. Subsequently, the beads were washed three times with 500 μl wash buffer and eluted twice with 50 μl elution buffer containing 20 mM Tris-HCl (pH 7.4), 10 mM EDTA, 300 mM imidazole, and 1% DDM. 20 μl of the eluted fractions were mixed with 5× SDS sample buffer and separated on a 8% SDS-PAGE gel. EGFP-tagged proteins were visualized using a Typhoon Trio fluorescence scanner (GE Healthcare).

### Electrophysiological recordings in Xenopus laevis oocytes

*Xenopus laevis* oocytes were obtained from Ecocyte Bioscience (Dortmund, Germany) or by partial ovariectomy (animal experiment approval ROB-55.2–2532.Vet_03-19–77) from frogs anesthetized in 0.2% tricaine (MS222). Oocytes were dissociated for 1–2 h at room temperature using collagenase type II (1.0 mg/mL; Nordmark Biochemicals, Germany) in Ca^2+^-free OR2 (90 mM NaCl, 1 mM KCl, 2 mM MgCl_2_, 5 mM HEPES, pH 7.4) solution and defolliculated by gentle agitation. Stage V–VI oocytes were selected and maintained at 16°C in sterile-filtered ND96 solution (96 mM NaCl, 2 mM KCl, 1 mM CaCl_2_, 1 mM MgCl_2_, 5 mM HEPES, pH 7.4) supplemented with 5 µg/mL gentamicin. Oocytes were injected with 50 nL cRNA (0.1–0.5 µg/µL) using a Nanoject II microinjector (Drummond Scientific, USA). For multimeric receptors, subunits were co-injected at a 1:1 ratio unless stated otherwise. Injected oocytes were incubated for 2–4 d prior to recording. Two-electrode voltage-clamp (TEVC) recordings were performed at a holding potential of −70 mV using a Turbo Tec-05X amplifier and CellWorks software 6.2.1 (npi electronic, Germany). Glass microelectrodes were filled with 3 M KCl and had resistances < 1 MΩ. Currents were filtered at 200 Hz and digitized at 400 Hz. The recording solution ND96, with or without 100 μM ACh, was automatically applied via a custom-made magnetic valve system combined with a manifold mounted closely above the oocyte, thus allowing a fast (<300 ms) and reproducible solution exchange. For generation of dose inhibition curves, agonist pulses (2 s) were applied in 4-min intervals (1 min with superfusion and 3 min static bath). Stable responses were defined as three consecutive responses differing by less than 10%. Then α-bungarotoxin (α-Btx) was applied into the static bath from a 10× stock solution and incubated for 3 min prior to another agonist application. Current responses were normalized to control responses recorded before inhibitor exposure. To generate agonist dose response curves, stable current responses were established by application of 2 s pulses of a reference concentration of (30 μM ACh, 1 μM for Neonicotinoids) in 3 min intervals. Then, the reference concentration and increasing test concentrations were alternatingly applied. Each test concentration was normalized to the mean of the preceding and following reference concentrations. Dose−response analyses were performed with GraphPad Prism version 10.6.1, and dose−response curves were fit to the data using the inbuilt Hill equation normalized response = (bottom + (top − bottom)/(1 + 10^((logIC_50_ − logc) × nH))) for antagonists and normalized response = (bottom + (top − bottom)/(1 + 10^((logEC_50_ − logc) × nH))) for agonists with n_H_ = Hill slope, c = concentration (μM), and bottom and top constrained to 0 and 1, respectively.

#### Statistical analysis

All data are shown as mean ± SD. Concentration–response, and inhibition data were analyzed by nonlinear regression using a four-parameter logistic model in GraphPad Prism 10.6.1. EC_50_ and IC_50_ values are reported with 95% confidence intervals. Statistical significance was assessed using unpaired Welch’s t-tests. Differences were considered statistically significant at *p* < 0.05. Significance levels are indicated as follows: *p < 0.05, **p < 0.01. For concentration–response curves (DRCs), electrophysiological recordings were obtained from 4–6 oocytes derived from two independent *Xenopus laevis* frogs, unless stated otherwise.

## Supplementary Material

Supplemental Material

Sequences Am a5 subuit.pdf

## Data Availability

The authors confirm that the data supporting the findings of this study are available within the article or its supplementary materials.
