# Supplementary material for: Functional expression of heteromeric insect/human nicotinic acetylcholine receptor chimeras without accessory proteins
Source: Channels (Austin). 2026 Oct 21;20(1):2736911. doi: 10.1080/19336950.2026.2736911 (PMC13690283; doi:10.1080/19336950.2026.2736911)
Supplement: Supplementary file 1 [file KCHL_A_2736911_SM2985.pdf]

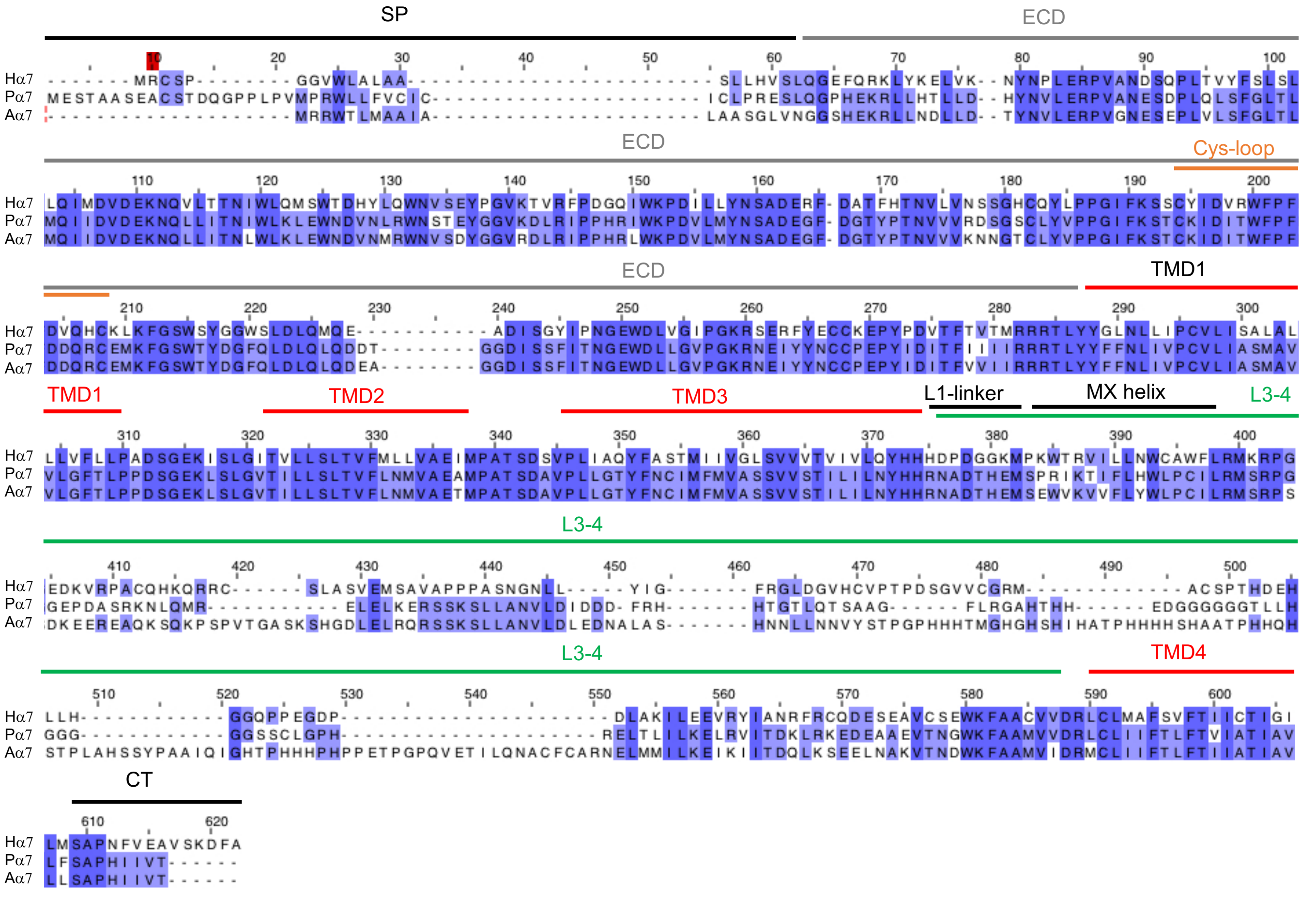

Supplementary Figure S1. Multiple sequence alignment of human cockroach and bee α7 nAChR subunits. Amino acid sequences of human α7 (Hα7), *Periplaneta americana* α7 (Paα7), and *Apis mellifera* α7 (Amα7) subunits were aligned using MAFFT. Amino acids that are present in all three species and in two species are highlighted in blue and purple, respectively. Coloured lines and letters indicate different protein domains.

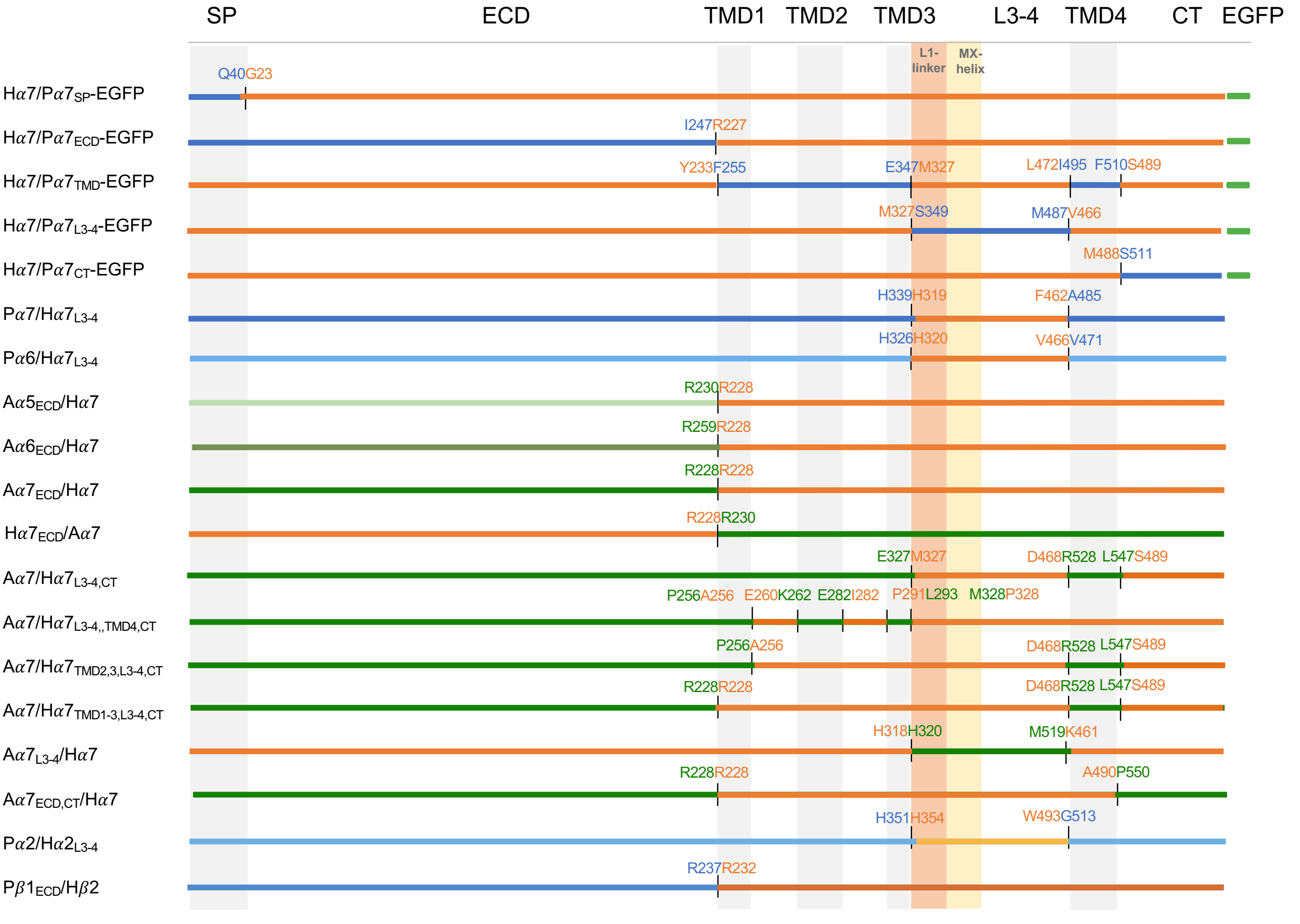

Supplementary Figure S2. Schematic overview of human/insect nAChR chimeras generated in this study. Domain architecture of chimeric nAChR constructs derived from human α7 (orange), *Periplaneta americana* α1, α2, α6, α7 and β1 (blue), and *Apis mellifera* α5, α6 and α7 (green) subunits. Chimeras were generated by exchanging the signal peptide (SP), extracellular domain (ECD), transmembrane domains (TMD1–TMD4), intracellular Loop (L3-4), and C-terminal region (CT). Numbers indicate the amino acid positions of the boundaries of the inserted domains and correspond to the residue numbering of the inserted subunit.

Supplementary Table T1. Accession numbers of nAChR subunits used for sequence alignment, phylogenetic analysis, and chimera design in this study.

Species	Subunit	Gene name	GenBank Accession	UniProt Accession
Periplaneta americana	α1	Pα1	JQ585634	R4I597
Periplaneta americana	α2	Pα2	KP725464	A0A0K1U4H6
Periplaneta americana	β1	Pβ1	MW201213	A0A7T5Y1L3
Periplaneta americana	α6	Pα6	KP725465	G9CU48
Periplaneta americana	α7	Pα7	MK790056.1	A0A5P8U3L1
Apis mellifera	α5	Aα5	KJ939592.1	A0A0H3V1C5
Apis mellifera	α6	Aα6	DQ026036	A0A7M6UC05
Apis mellifera	α7	Aα7	AY500239.1	A0A7M6UUB9
Homo sapiens	α2	Hα2	NM_000742	Q15822
Homo sapiens	β2	Hβ2	NM_000748	P17787
Homo sapiens	α7	Hα7	NM_000746	P36544

GenBank DNA accession numbers and UniProt protein accession for nAChR subunits from *Periplaneta americana*, *Apis mellifera*, and *Homo sapiens* included in this study.

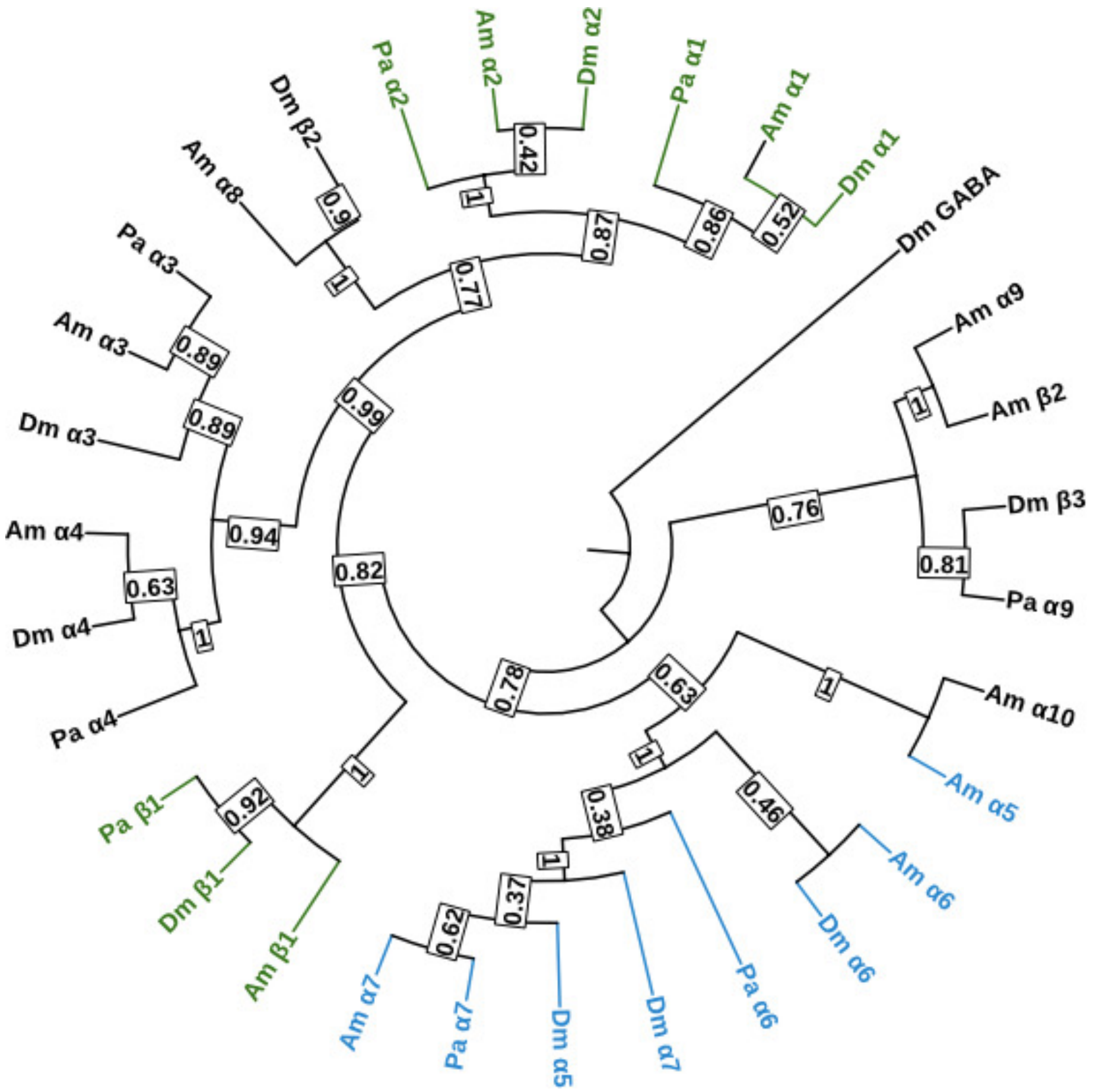

Supplementary Figure S3: Phylogenetic tree of insect nAChR subunits from *Periplaneta americana*, *Apis mellifera,* and *Drosophila melanogaster.*

To analyze evolutionary relationships between insect nAChR subunits and guide the selection of candidate receptor assemblies, the amino acid sequences were retrieved from the UniProt database *Apis mellifera* α1 (A0A7M7MMZ9), α2 (A0A0H3V1C5), α3 (A0EIZ2), α4 (A0EIZ3), α8 (Q8MUR0), α9 (A0EIZ7), α10 (XP_392070), β1 (A0EIZ8), and β2 (A0EIZ9); *Drosophila melanogaster* α1 (P09478), α2 (P17644), α3 (Q9W3G6), α4 (A8JNX5), α5 (Q7KT97), α6 (Q9VL79), α7 (Q9VWI9), β1 (P04755), and β3 (Q9VPQ8); *Periplaneta americana*, α3 (A0A0K0QSP6), α4 (H6TY11), α9 (A0A7T5Y176) (For the other Numbers se Table S1). The γ-aminobutyric acid receptor subunit GBR (P25123) from *D. melanogaster* was included as an outgroup. Protein sequences were aligned using MAFFT version 7 (1). Poorly aligned and phylogenetically uninformative regions were removed using BMGE (Block Mapping and Gathering with Entropy) (2). Phylogenetic reconstruction was performed using a maximum likelihood approach implemented in PhyML 3.0 (3) through the NGPhylogeny.fr phylogenetic analysis platform (4). Branch support values were assessed using 100 non-parametric bootstrap replicates, and bootstrap values were mapped onto the consensus tree. The resulting phylogenetic tree was visualized and annotated using the Interactive Tree of Life (iTOL) platform (5). The analysis resolved distinct clades corresponding to α1/α2-like and α5/α6/α7-like receptor groups across insect species.

(1) Katoh and Standley, 2013 ***Molecular Biology and Evolution***
(2) Criscuolo and Gribaldo, 2010 ***BMC Evolutionary Biology***
(3) Guindon et al., 2010 ***Systematic Biology***
(4) Lemoine et al., 2019 ***Nature***
(5) Letunic and Bork, 2024 ***Nucleic Acid Research***

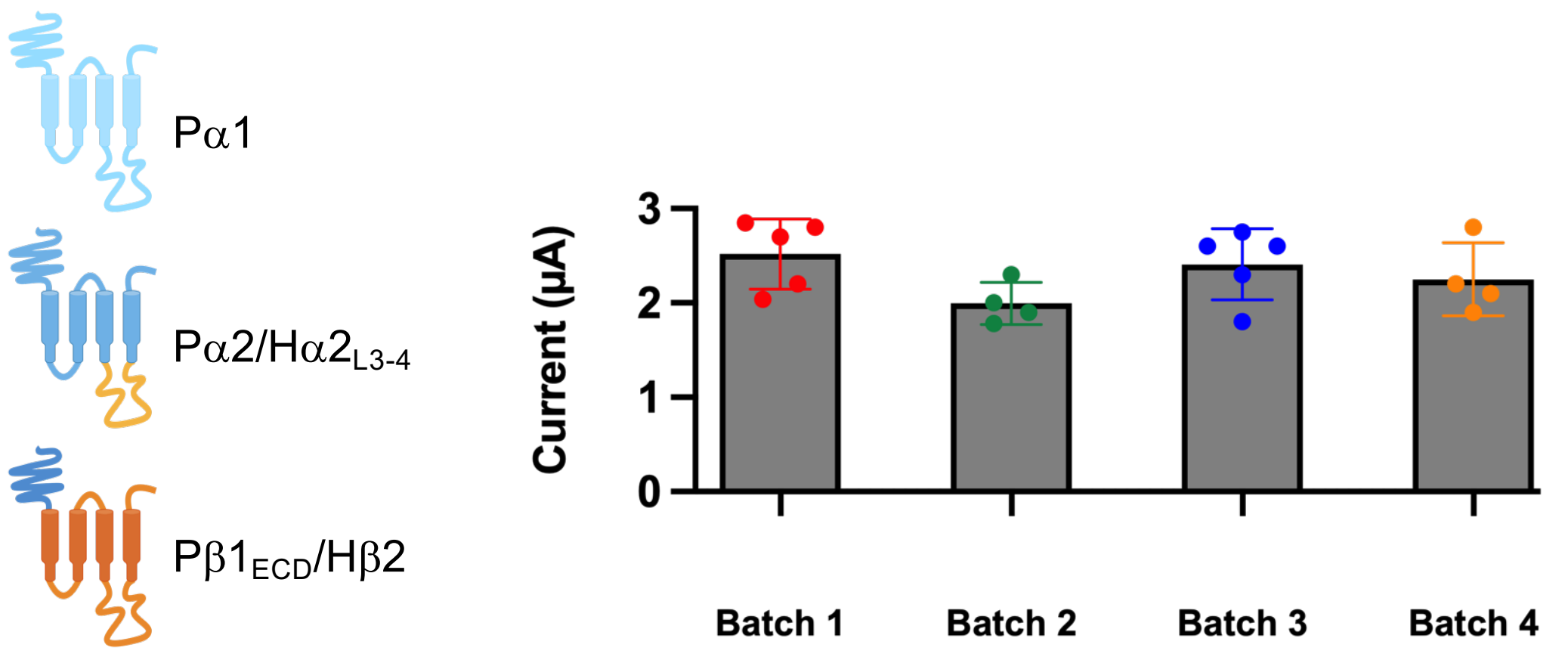

Supplementary Figure S4. Comparison of ACh-induced current amplitudes obtained from cRNA-injected *Xenopus laevis* oocytes of different donor animals. Oocytes were co-injected with cRNAs encoding the indicated nAChR subunits: Pα1, Pα2/H$\alpha 2_{L3\text{-}4}$, and P$\beta 1_{ECD}$/Hβ2 and currents were activated with 100 µM ACh. n=4-5 responses from individual oocytes of one batch were recorded and each colour represents a different donor animal (N=4). Means ± SD are shown. A one-way ANOVA did not reveal evidence of systematic differences between batches [$F(3,14)=1.835, p=0.1871$] and there was no evidence of variance heterogeneity (Brown–Forsythe test, $p=0.9152$; Bartlett's test, $p=0.8104$).
