# Supplementary material for: Functional expression of heteromeric insect/human nicotinic acetylcholine receptor chimeras without accessory proteins
Source: Channels (Austin). 2026 Oct 21;20(1):2736911. doi: 10.1080/19336950.2026.2736911 (PMC13690283; doi:10.1080/19336950.2026.2736911)
Supplement: Supplementary file 2 [file KCHL_A_2736911_SM6943.pdf]

Original sequence of the *Apis mellifera* α5 plasmid from 30.06.2022

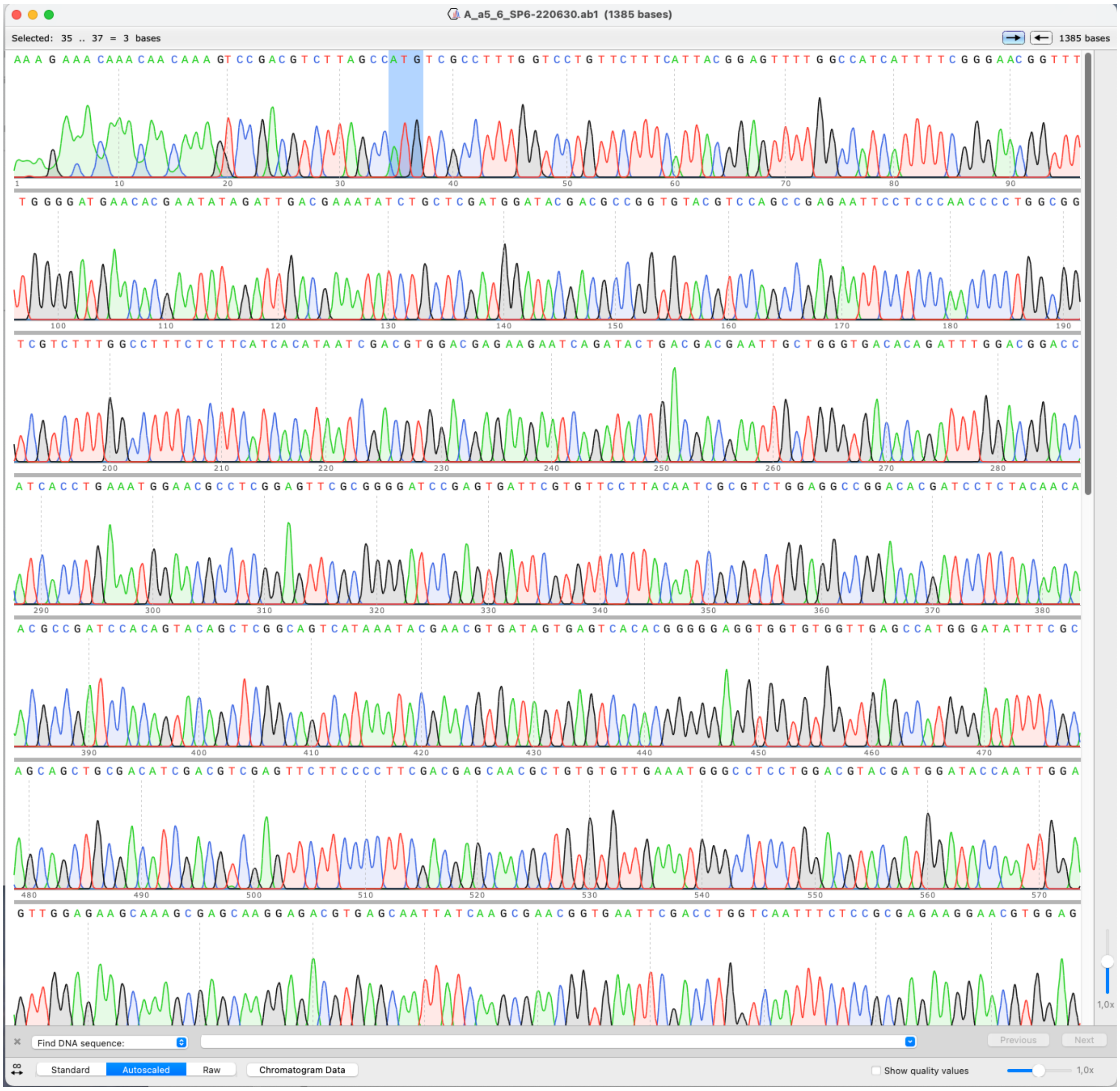

Re-sequencing of the *Apis mellifera* α5 plasmid from 27.07.2026

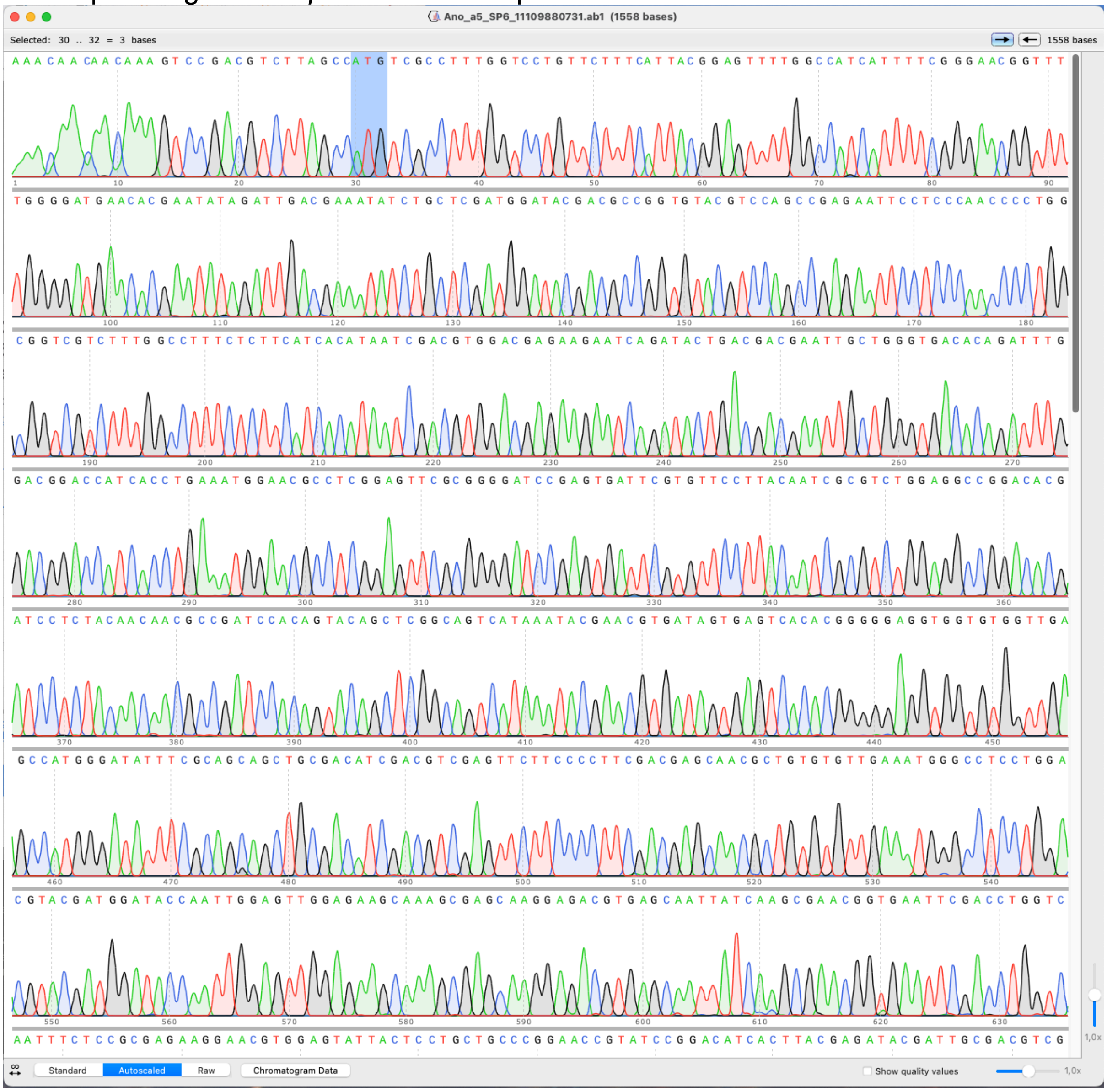

Alignment of the sequence obtained from the above plasmid with with GenBank KJ939592.1: coding region 1443/1443 bp = 100 % identity, 0 mismatches

```
KJ939592.1    1 ATGTCGCCTTTGGTCCTGTTCTTTCATTACGGAGTTTTGGCCATCATTTTCGGGAACGGTTTTGGGGATGAACACGAATATAGATTGACGAAATATCTGCTCGATGGATACGACGCCGGTGTACGTCCAGCCGAGAATTCCTCCCAACCC  150
                ||||||||||||||||||||||||||||||||||||||||||||||||||||||||||||||||||||||||||||||||||||||||||||||||||||||||||||||||||||||||||||||||||||||||||||||||||||||||||
Our seq.      1 ATGTCGCCTTTGGTCCTGTTCTTTCATTACGGAGTTTTGGCCATCATTTTCGGGAACGGTTTTGGGGATGAACACGAATATAGATTGACGAAATATCTGCTCGATGGATACGACGCCGGTGTACGTCCAGCCGAGAATTCCTCCCAACCC  150

KJ939592.1  151 CTGGCGGTCGTCTTTGGCCTTTCTCTTCATCACATAATCGACGTGGACGAGAAGAATCAGATACTGACGACGAATTGCTGGGTGACACAGATTTGGACGGACCATCACCTGAAATGGAACGCCTCGGAGTTCGCGGGGATCCGAGTGATT  300
                ||||||||||||||||||||||||||||||||||||||||||||||||||||||||||||||||||||||||||||||||||||||||||||||||||||||||||||||||||||||||||||||||||||||||||||||||||||||||||
Our seq.    151 CTGGCGGTCGTCTTTGGCCTTTCTCTTCATCACATAATCGACGTGGACGAGAAGAATCAGATACTGACGACGAATTGCTGGGTGACACAGATTTGGACGGACCATCACCTGAAATGGAACGCCTCGGAGTTCGCGGGGATCCGAGTGATT  300

KJ939592.1  301 CGTGTTCCTTACAATCGCGTCTGGAGGCCGGACACGATCCTCTACAACAACGCCGATCCACAGTACAGCTCGGCAGTCATAAATACGAACGTGATAGTGAGTCACACGGGGGAGGTGGTGTGGTTGAGCCATGGGATATTTCGCAGCAGC  450
                ||||||||||||||||||||||||||||||||||||||||||||||||||||||||||||||||||||||||||||||||||||||||||||||||||||||||||||||||||||||||||||||||||||||||||||||||||||||||||
Our seq.    301 CGTGTTCCTTACAATCGCGTCTGGAGGCCGGACACGATCCTCTACAACAACGCCGATCCACAGTACAGCTCGGCAGTCATAAATACGAACGTGATAGTGAGTCACACGGGGGAGGTGGTGTGGTTGAGCCATGGGATATTTCGCAGCAGC  450

KJ939592.1  451 TGCGACATCGACGTCGAGTTCTTCCCCTTCGACGAGCAACGCTGTGTGTTGAAATGGGCCTCCTGGACGTACGATGGATACCAATTGGAGTTGGAGAAGCAAAGCGAGCAAGGAGACGTGAGCAATTATCAAGCGAACGGTGAATTCGAC  600
                ||||||||||||||||||||||||||||||||||||||||||||||||||||||||||||||||||||||||||||||||||||||||||||||||||||||||||||||||||||||||||||||||||||||||||||||||||||||||||
Our seq.    451 TGCGACATCGACGTCGAGTTCTTCCCCTTCGACGAGCAACGCTGTGTGTTGAAATGGGCCTCCTGGACGTACGATGGATACCAATTGGAGTTGGAGAAGCAAAGCGAGCAAGGAGACGTGAGCAATTATCAAGCGAACGGTGAATTCGAC  600

KJ939592.1  601 CTGGTCAATTTCTCCGCGAGAAGGAACGTGGAGTATTACTCCTGCTGCCCGGAACCGTATCCGGACATCACTTACGAGATACGATTGCGACGTCGACCGATGTTTTACGTCTTCAATCTGATACTTCCATGCATACTCATCAACAGTGTC  750
                ||||||||||||||||||||||||||||||||||||||||||||||||||||||||||||||||||||||||||||||||||||||||||||||||||||||||||||||||||||||||||||||||||||||||||||||||||||||||||
Our seq.    601 CTGGTCAATTTCTCCGCGAGAAGGAACGTGGAGTATTACTCCTGCTGCCCGGAACCGTATCCGGACATCACTTACGAGATACGATTGCGACGTCGACCGATGTTTTACGTCTTCAATCTGATACTTCCATGCATACTCATCAACAGTGTC  750

KJ939592.1  751 GCCCTGTTGGTATTCTACGTGCCGTCTGAATCGGGGGAAAAAGTCACCCTCGGGATTTCGGCCCTCCTCTCTATGACGGTCTTCCTAATGACTATTCGCGAGTCACTGCCGCCCACGGAGAAAACACCATTAATAAGTCTTTATTACGGT  900
                ||||||||||||||||||||||||||||||||||||||||||||||||||||||||||||||||||||||||||||||||||||||||||||||||||||||||||||||||||||||||||||||||||||||||||||||||||||||||||
Our seq.    751 GCCCTGTTGGTATTCTACGTGCCGTCTGAATCGGGGGAAAAAGTCACCCTCGGGATTTCGGCCCTCCTCTCTATGACGGTCTTCCTAATGACTATTCGCGAGTCACTGCCGCCCACGGAGAAAACACCATTAATAAGTCTTTATTACGGT  900

KJ939592.1  901 GTCAGTATATGCCTGGTGACATTCGCTTCAGCGTTAGCGGTGGTCACGTTGAATCTTCATCACCGCGGTGTTCGTGGTACCAGAGTACCGGGTATCGTTAGATCGTTGGTGTTGGACAAGCTCGCAAGGATAGTGCTCCTCAACTTTCAA 1050
                ||||||||||||||||||||||||||||||||||||||||||||||||||||||||||||||||||||||||||||||||||||||||||||||||||||||||||||||||||||||||||||||||||||||||||||||||||||||||||
Our seq.    901 GTCAGTATATGCCTGGTGACATTCGCTTCAGCGTTAGCGGTGGTCACGTTGAATCTTCATCACCGCGGTGTTCGTGGTACCAGAGTACCGGGTATCGTTAGATCGTTGGTGTTGGACAAGCTCGCAAGGATAGTGCTCCTCAACTTTCAA 1050

KJ939592.1 1051 GAGGAGAGAAGATCGGAACCGGTCGAACCGCCGAGAAGAAAAAACAATTGTCCGCTGCACTGCAAACCGGAGCTGGGCCAATCGCAATCGTCGCCCAAGTTCGTAGCCAGAAGGGAGGAGAGTAATAGCAGCAGTCCTAGTTTAGATCTT 1200
                ||||||||||||||||||||||||||||||||||||||||||||||||||||||||||||||||||||||||||||||||||||||||||||||||||||||||||||||||||||||||||||||||||||||||||||||||||||||||||
Our seq.   1051 GAGGAGAGAAGATCGGAACCGGTCGAACCGCCGAGAAGAAAAAACAATTGTCCGCTGCACTGCAAACCGGAGCTGGGCCAATCGCAATCGTCGCCCAAGTTCGTAGCCAGAAGGGAGGAGAGTAATAGCAGCAGTCCTAGTTTAGATCTT 1200

KJ939592.1 1201 GGAAAAGAAGGTGGCCTTGAGGCGCAATGGTCACGCGTGCTAGGGAGGGTGCACGCGACTATAGAGAGAAACGAGAAACGGCTCGCGGAGCAGGATCGTCGCGAGCGAATGGAATTTGATTGGAAACAGGTCGCGTTGGTCAGCGATCGA 1350
                ||||||||||||||||||||||||||||||||||||||||||||||||||||||||||||||||||||||||||||||||||||||||||||||||||||||||||||||||||||||||||||||||||||||||||||||||||||||||||
Our seq.   1201 GGAAAAGAAGGTGGCCTTGAGGCGCAATGGTCACGCGTGCTAGGGAGGGTGCACGCGACTATAGAGAGAAACGAGAAACGGCTCGCGGAGCAGGATCGTCGCGAGCGAATGGAATTTGATTGGAAACAGGTCGCGTTGGTCAGCGATCGA 1350

KJ939592.1 1351 GCGCTTCTTTGCGTTTTTTTTTTGACCACGATCGTCAGCACCACGGTCATCCTGTGTGGTTCTCCGCCAAGCGCGAACATTGCCAAAGAGGGT------------------------TAA 1446
                |||||||||||||||||||||||||||||||||||||||||||||||||||||||||||||||||||||||||||||||||||||||||||||                        |||
Our seq.   1351 GCGCTTCTTTGCGTTTTTTTTTTGACCACGATCGTCAGCACCACGGTCATCCTGTGTGGTTCTCCGCCAAGCGCGAACATTGCCAAAGAGGGTCATCACCATCACCATCACCATCACTAA 1470
```

Dashes (-) mark the C-terminal His$_8$ purification tag present in our sequence; it is absent from KJ939592.1 and does not alter the α5 coding sequence.
